# Unproven stem cell-based interventions marketed as treatments in Panama: An urgent call for enforcement actions

**DOI:** 10.1016/j.stemcr.2026.103017

**Published:** 2026-07-23

**Authors:** Mairim Alexandra Solis, Magalys Quintana, Jesica E. Candanedo P., Rita Inés Trujillo-Sagel, Lynn Chambonnet, Sulgeis Lasso, Members of the National Research Bioethics Committee

**Affiliations:** 1 Gorgas Memorial Institute for Health Studies, Panama, Republic of Panama; 2 National Research Bioethics Committee, Panama, Republic of Panama; 3 Caja de Seguro Social, Panama, Republic of Panama; 4 Secretaría Nacional de Ciencia, Tecnología e Innovación (SENACYT), Panama, Republic of Panama; 5 Ministry of Health, Panama, Republic of Panama; 6 Sistema Nacional de Investigación (SNI-AIP), Panama, Republic of Panama

## Abstract

The marketing of unproven stem cell-based interventions raises public health, ethical, and regulatory challenges. This article examines online marketing in Panama, identifies new businesses, and argues that lack of enforcement has contributed to their expansion. Urgent actions are needed to mitigate this market, protect patients, and generate evidence-based treatments.

## Introduction

For over a decade, clinics across the globe have been establishing themselves in Latin America to offer unproven stem cell-based interventions (SCI) for a wide range of diseases and conditions, despite lacking robust evidence of safety and efficacy (Turner et al., 2023). Patients travel to countries with lenient regulations for these treatments, a phenomenon known as “stem cell tourism”, despite the potential harm. Severe adverse events following unproven SCI, including thromboembolism, fibrosis, tumors, severe vision loss, and death, have been extensively reported (Baranovskii et al., 2022; Bauer et al., 2018). Several studies have identified businesses in various countries offering unproven SCI. The first business identified in Panama was in 2013, when the Institute for Cellular Medicine, previously located in India, Mexico, and Costa Rica, was set up in Panama (Berger et al., 2016; Connolly et al., 2014; Ogbogu et al., 2013; Turner et al., 2023). During that period, numerous advertisements were published in local newspapers, travel magazines, and on social media, using testimonials from celebrities of the “treatments” received. The serious regulatory gap, together with increased marketing of unproven SCI, highlighted the need for a new regulatory framework, leading to the adoption of Executive Decree No. 179 of 8 June 2018 (Decree 179) by the Ministry of Health of Panama (MoHP). Despite this action, the marketing of unproven SCI outside a research context or an approved hematological treatment remains a concern in Panama. Regulatory actions in Australia and Canada have led to a notably decrease in unproven SCI market size, a shift in the types of biologics advertised, and a curb of marketing claims (Ikonomou et al., 2024); an approach that could be adopted. In this article, we examine online marketing practices of businesses currently offering unproven SCI and discuss the limitations in the compliance of the regulatory framework in Panama. We also call for collective efforts in Panama to strengthen stem cell research and its clinical translation and urgent enforcement actions by national regulatory bodies to restrain these unscrupulous businesses and more effectively regulate this health market.

### Regulatory framework for stem cell research and clinical translation in Panama

**Figure 1:**
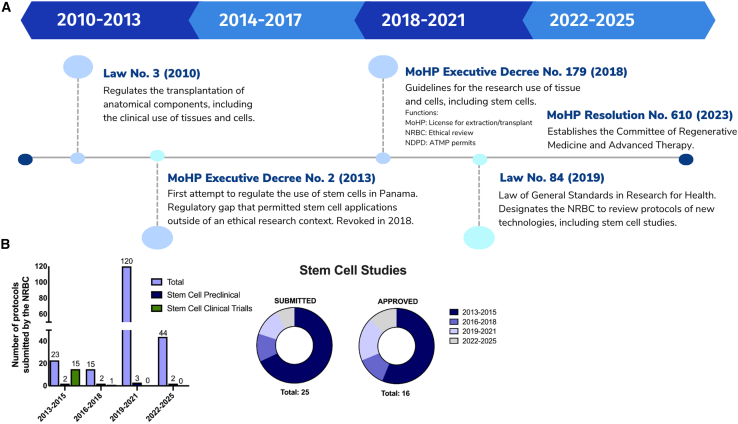
Timeline of the regulatory framework for the use of stem cells in Panama and the NRBC role in stem cell research (A) Timeline of regulations regarding stem cell research in Panama; (B) Total number of stem cell research protocols submitted and approved per year. MoHP, Ministry of Health of Panama; NRBC, National Research Bioethics Committee; NDPD, National Directorate of Pharmacy and Drugs.

Law 3 of 2010 in Panama regulates the transplantation of anatomical components, including human tissue and cells (Law No. 3 of February 8, 2010). However, stem cell research and the development of advanced therapy medicinal products were not specifically addressed. The first attempt to regulate the use of stem cells in Panama was the Executive Decree of the MoHP No. 2 of 21 January 2013. This action provided guidelines for the establishment of clinical centers authorized to provide stem cell interventions. However, this decree did not clearly distinguish between approved and unproven SCI, thus permitting stem cell applications outside of an ethical research context which was in opposition to Law 3 of 2010. This ambiguity, together with an increase in the marketing of unproven SCI, prompted the approval of Decree 179 (Executive Decree No. 179 of June 8, 2018), which revoked the previous decree and established new guidelines for clinical translation of stem cell research. The Interinstitutional Committee of Regenerative Medicine and Advanced Therapies of the MoHP was established, comprising members from the MoHP, National Research Bioethics Committee, National Transplant Committee, research institutions, universities, and hospitals (Resolution No. 610 of August 7, 2023) (Figure 1A). The purpose of this Committee was to establish, coordinate and update related policies in Panama.

Decree 179 specifically aligned with recommendations made by the International Society for Stem Cell Research (ISSCR) for the safe and efficacious translation of SCIs (ISSCR, 2016). These guidelines denoted that the only currently accepted clinical practice related to the use of stem cells is the transplantation of hematopoietic tissue and cells, autologous and allogeneic, for hematological diseases. Any other stem cell use should be viewed as experimental and be subjected to evaluation in clinical trials. Decree 179 states that centers interested in conducting research with human tissues and cells, including stem cells of any tissue source and type, except those derived from embryos, must apply to the MoHP for a license for the extraction and transplantation of anatomical components, including hospitals or banks for hematopoietic tissues or cell preparation, preservation, and storage (Law No. 3 of February 8, 2010). Currently, only four centers in Panama have an active license for hematopoietic stem cell extraction, transplantation, and therapeutic treatment: Instituto Oncológico Nacional, the Complejo Hospitalario Dr. Arnulfo Arias Madrid, the Hospital del Niño Dr. José Renán Esquivel, and The Panama Clinic. The rigorous review of license application has apparently reduced the legal establishment of centers with unethical clinical practices, a notable regulatory achievement. Stem cells used in clinical trials are considered advanced therapy medicinal products (ATMPs) regulated by the National Directorate of Pharmacy and Drugs (NDPD) of the MoHP. The NDPD coordinates their use for research and issues ATMP Use Permit (Executive Decree No. 179 of June 8, 2018; Law No. 419 of February 1, 2024). However, local NDPD processes and capacities for implementing and issuing ATMP permits require strengthening to enable effective regulatory oversight.

Another crucial aspect in the regulation of research involving human tissues and cells in Panama has been, since 2013, the requirement that any stem cell-related study be submitted to, reviewed and approved by the National Research Bioethics Committee of Panama (NRBC), with the National Transplant Committee providing advisory guidance for clinical trials. The NRBC’s role was strengthened in 2019 reaffirming its function of conducting ethics reviews of new technologies, such as stem cell research (Law No. 84 of May 14, 2019) (Figure 1A). Between 2013 and 2025, 202 research protocols were submitted to the NRBC. Among these, 25 were stem cell-related pre-clinical and clinical trials, of which 16 were approved, 3 rejected, and 6 closed by the NRBC, representing a 64% approval rate (Figure 1B). The submission of protocols peaked in 2020, in the context of resolution 373 of 2020, later replaced by resolution no. 400 of 2021, which designated the NRBC to review all COVID-19-related research. Since 2018, no stem cell-related clinical trial protocols have been submitted to the NRBC, nor have ATMP applications been submitted to the NDPD, possibly reflecting avoidance of the regulatory review process. In contrast, advertising of unproven SCI has recently increased, as several businesses marketing these interventions on social media have been identified.

### Recent expansion of businesses offering unproven SCI

**Figure 2:**
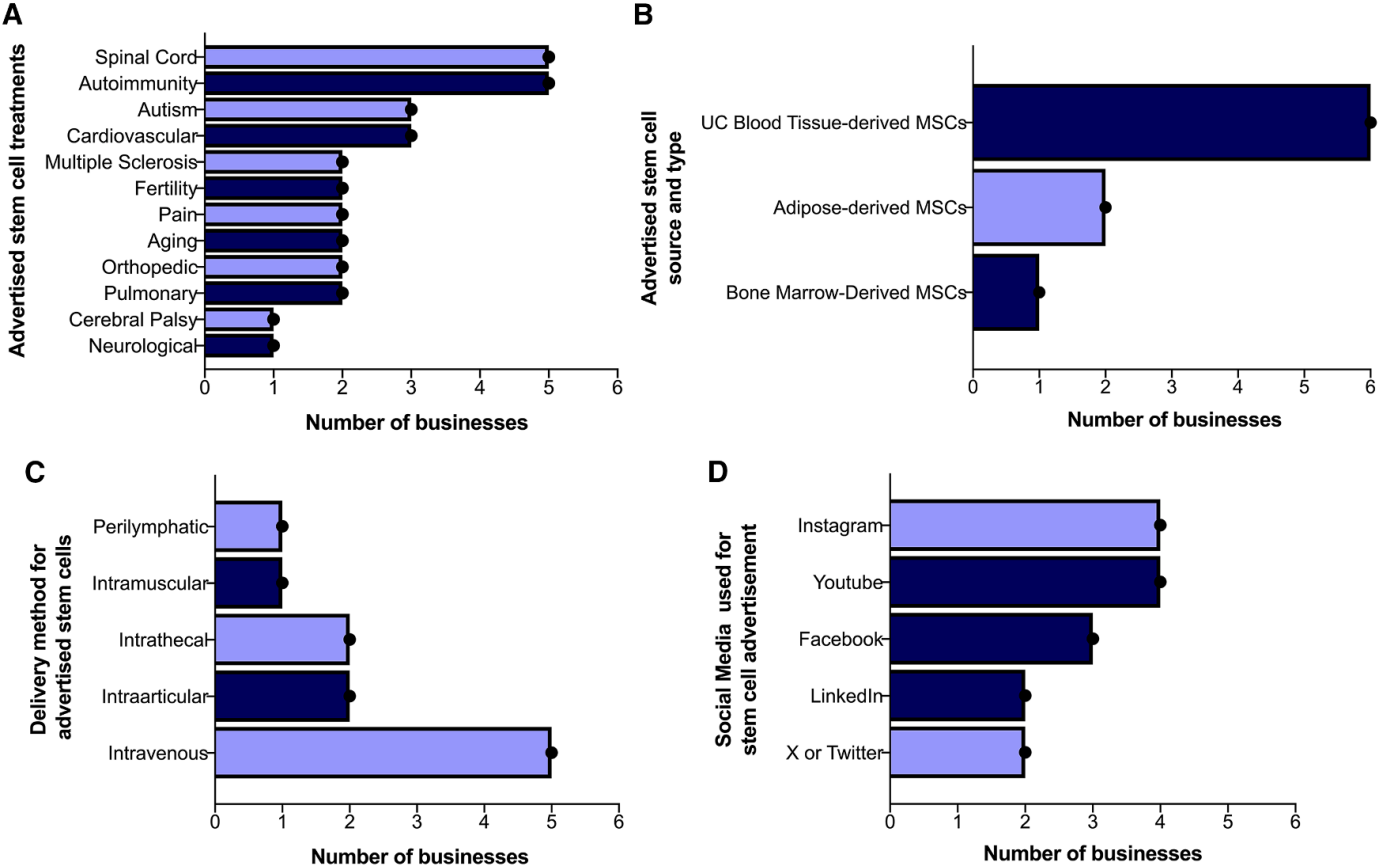
Businesses marketing unproven SCI in Panama (A) Unproven SCI marketed as treatments by businesses; (B) stem cell source and type used by businesses for unproven SCI; (C) delivery method used by businesses to administer unproven SCI; (D) social media used by businesses to advertise unproven SCI. UC, umbilical cord; MSCs, mesenchymal stem cells.

To examine the extent of unproven SCI currently offered in Panama, websites of local businesses marketing such interventions were searched, identified and their content analyzed in October 2025. Search terms included “stem cell clinic in Panama”, “stem cell center in Panama”, “stem cell treatment in Panama”, and “social media of stem cell clinic in Panama”. Data included “treatment” details, stem cell source and type, administration method, social media used for advertising, pricing, tourism package offered, and research claims (Figure 2). Patient testimonials were also identified from business websites and social media. A total of six businesses were found to be marketing unproven SCI in Panama, five were more than previously reported (Connolly et al., 2014; Turner et al., 2023). Each business advertised between six and ten unproven SCI for a range of conditions including spinal cord injury, autoimmunity, cardiovascular diseases, autism, multiple sclerosis, fertility, neurological, pulmonary, orthopedic, age-related, pain, and cerebral palsy (Figure 2A). All of these businesses marketed on their websites use of mesenchymal stem cells (MSCs) derived from either umbilical cord blood or tissue; two businesses also stated use of adipose-derived MSC; and one bone marrow-derived MSC (Figure 2B). The administration method was intravenous (83%), intrathecal or intra-articular (33%), and either intramuscular or perilymphatic for one center (Figure 2C). Prices ranged from $5,000 to $50,000. Social media platforms were embedded on their websites, including Instagram or YouTube (67%), Facebook, LinkedIn, or X/Twitter (33%) (Figure 2D). All businesses (100%) offered medical tourism packages, combining “treatment” with additional services, such as flights, accommodation, and local tours. More than 200 patient testimonials were identified, ranging from 13 to more than 170 per business.

### Current challenges, actions to date, and recommendations

Advertising of unauthorized medical services or medicines with false attributes is prohibited and subject to sanctions (Law No. 66 of November 10, 1947). The NRBC, in its role of reporting regulatory noncompliance, has consistently notified the MoHP these businesses’ violations of applicable laws and regulations. Between 2015 and 2016, the NRBC identified and reported multiple advertisements for unproven stem cell interventions in local newspapers and travel magazines, leading to four formal notifications to the MoHP. More advertisements continued to appear through 2017 and 2018 in local newspapers, frequently featuring testimonials from public figures as part of marketing strategies, prompting an additional formal notification. From 2019 to 2022, no irregular advertisements were detected, likely due to regulations and COVID-19 lockdown. Advertisements were again detected in 2023 in a local newspaper, a medical tourism magazine, and websites, leading to a new formal notification in 2024 and another in 2025 that compiled all six unaddressed notifications of irregularities concerning businesses offering unproven SCI.

The lack of enforcement by regulatory bodies, such as warning letters or regulatory sanctions against businesses advertising unproven SCI, has likely facilitated the growth of this unproven industry in Panama. Current legislation defines the sanctions for non-compliance with the decree, detailing violations, sanctions, and procedures for MoHP enforcement (Law No. 3 of February 8, 2010). Despite having established an adverse event reporting mechanism, no complaints have been documented, underscoring the need for an accessible platform for reporting these events.

There have been several attempts from some of these businesses to modify stem cell regulations in Panama, aimed at legalizing the commercialization of unproven MSC-based interventions outside of research contexts and ATMP procedures, potentially posing public health risks. In 2020, a bill was proposed seeking to authorize stem cell use in clinical trials without prior approval from the NDPD. In 2022, a draft decree was introduced to enable the commercialization of MSC-based interventions based solely on safety data and preliminary efficacy evidence derived from Phase I clinical trials. In 2023, another draft decree sought to authorize the therapeutic use of unproven MSC-based interventions. The NRBC recommended against each of these, a position supported by the MoHP, thus, none of these initiatives were approved.

Such potential life-threatening situations for people undertaking unproven SCIs is a clear violation of the national regulations and the corresponding enforcement actions through warning letters or sanctions should be imposed, as stated in Decree 179. Social media has been used for marketing unproven SCI by individuals who are not scientific or medical experts, spreading misinformation to patients (Grundtvig and Munsie, 2025). Scientific communication strategies must be implemented by legitimate stem cell researchers, ethicists, and related professionals to educate people about legitimate stem cell therapies and reach a wider audience. Regulatory bodies are advised to implement strategies to monitor and counteract the spread of misleading information on digital platforms, particularly social media. The ISSCR guidelines serve as a standard for regulatory formulation that promote an ethical, practical, appropriate, and sustainable initiative for stem cell research and the development of cell therapies that will improve human health and should be available for patients.

## Conclusions

The potential public health risks associated with unproven SCI demand prompt actions to reinforce the national regulatory framework to guarantee public health protection. Despite progress in regulating stem cell research and its clinical application in Panama, the increasing number of businesses marketing unproven SCI is concerning. Regulatory bodies should prioritize strict enforcement of warning letters or sanctions against businesses marketing unproven therapies. Accurate, scientific information regarding the validity and risks of stem cell applications is crucial for safeguarding the public, informing medical professionals, and protecting legitimate research advancements from unfounded claims. The positive effects of regulatory action taken by other countries in this area are clear, and this approach could be adopted to mitigate local unproven stem cell marketing and promote patient safety, while encouraging ethical and scientifically evidenced stem cell research that will open new possibilities in this promising field. It is time for regulatory bodies in Panama to take action.

## Acknowledgments

The authors would like to express their sincere gratitude to Prof. Megan Munsie from the 10.13039/100014555Murdoch Children’s Research Institute and the 10.13039/501100000987University of Melbourne for her insightful comments and feedback on this manuscript. This work was supported through the NRBC funds from the 10.13039/501100007061National Secretariat of Science, Technology and Innovation (SENACYT), and the 10.13039/501100017486Sistema Nacional de Investigación de Panamá (SNI, SENACYT) (SNI242-2022 and SNI2R2025-018 M.A.S.).

## Author contributions

M.A.S. conceptualized and drafted the first version of the manuscript. M.A.S, M.Q., and J.E.C.P. collected data. M.A.S., J.E.C.P., and R.I.T-S., written contribution. M.A.S., M.Q, J.E.C.P., R.I.T-S., L.C., and S.L., critically contributed to the intellectual content and in the drafting process. All authors, except for M.Q, are current members of the NRBC. M.Q. participated in the elaboration of this manuscript while Technical Secretary of the NRBC. All authors and other members of the NRBC approved the final version of the manuscript.

## Declaration of interests

The authors declare no competing interests.
